# COVID-19 Outbreak Related to PM_10_, PM_2.5_, Air Temperature and Relative Humidity in Ahvaz, Iran

**DOI:** 10.1007/s44229-022-00020-z

**Published:** 2022-11-30

**Authors:** Yusef Omidi Khaniabadi, Pierre Sicard, Bahram Dehghan, Hassan Mousavi, Saeid Saeidimehr, Mohammad Heidari Farsani, Sadegh Moghimi Monfared, Heydar Maleki, Hojat Moghadam, Pouran Moulaei Birgani

**Affiliations:** 1 Occupational and Environmental Health Research Center, Petroleum Industry Health Organization (PIHO), Ahvaz, Iran; 2 ARGANS, Sophia-Antipolis, France; 3 Family Health Research Center, Petroleum Industry Health Organization (PIHO), Ahvaz, Iran; 4 School of Medicine, Ahvaz Jundishapur University of Medical Sciences, Ahvaz, Iran; 5 Gachsaran Oil and Gas Production Company, National Iranian Oil Company, Gachsaran, Iran; 6 Department of Environmental Health Engineering, School of Public Health, Student Research Committee, Ahvaz Jundishapur University of Medical Sciences, Ahvaz, Iran

**Keywords:** COVID-19, PM_10_ and PM_2.5_, Temperature, Relative humidity, Petroleum Hospital

## Abstract

In this study, we assessed several points related to the incidence of COVID-19 between March 2020 and March 2021 in the Petroleum Hospital of Ahvaz (Iran) by analyzing COVID-19 data from patients referred to the hospital. We found that 57.5% of infected referrals were male, 61.7% of deaths by COVID-19 occurred in subjects over 65 years of age, and only 2.4% of deaths occurred in younger subjects (< 30 years old). Analysis showed that mean PM_10_ and PM_2.5_ concentrations were correlated to the incidence of COVID-19 (*r* = 0.547, *P* < 0.05, and *r* = 0.609, *P* < 0.05, respectively) and positive chest CT scans (*r* = 0.597, *P* < 0.05, and *r* = 0.541, *P* < 0.05 respectively). We observed that a high daily air temperature (30–51 °C) and a high relative humidity (60–97%) led to a significant reduction in the daily incidence of COVID-19. The highest number of positive chest CT scans were obtained in June 2020 and March 2021 for daily air temperature ranging from 38 °C and 49 °C and 11 °C and 15 °C, respectively. A negative correlation was detected between COVID-19 cases and air temperature (*r* =  − 0.320, *P* < 0.05) and relative humidity (*r* =  − 0.384, *P* < 0.05). In Ahvaz, a daily air temperature of 10–28 °C and relative humidity of 19–40% are suitable for the spread of coronavirus. The highest correlation with the number of COVID-19 cases was found at lag3 (*r* = 0.42) and at lag0 with a positive chest CT scan (*r* = 0.56). For air temperature and relative humidity, the highest correlations were found at day 0 (lag0). During lockdown (22 March to 21 April 2020), a reduction was observed for PM_10_ (29.6%), PM_2.5_ (36.9%) and the Air Quality Index (33.3%) when compared to the previous month. During the pandemic period (2020–2021), the annual mean concentrations of PM_10_ (27.3%) and PM_2.5_ (17.8%) were reduced compared to the 2015–2019 period.

**Supplementary Information:**

The online version contains supplementary material available at 10.1007/s44229-022-00020-z.

## Introduction

The COVID-19 outbreak globally progressed from Wuhan (China) in December 2019 to all regions of the world [15, 82]. The World Health Organization (WHO) confirmed more than 118,000 COVID-19 cases in 114 countries and 4291 deaths on 11 March 2020 and declared the beginning of a new global pandemic (World Health Organization, 2020). The number of new cases increased sharply worldwide with 3,942,907 infected people and 271,646 deaths reported in May 2020 in more than 200 countries [82]. In Iran, approximately 9,000 COVID-19 cases and 354 deaths were reported on 11 March 2020 (https://behdasht.gov.ir). The first COVID-19 case in Iran was reported in Qom county on 19 February 2020 and on 23 February 2020 in Ahvaz [32, 68]. In Iran, the highest number of daily infected cases (14,051) and deaths (486) were reported on 27 and 16 November 2020, respectively (https://worldometers.info). With the virus spreading, the Iranian government started programs for reducing fatalities; one of these was lockdown. The first lockdown started on 22 March 2020. Hence, a reduction of air pollution was observed due to limited anthropogenic activities [58, 64].

COVID-19 is well known as an acute respiratory disease leading to pneumonia with symptoms including fever, cough and dyspnea [39] with a fatality rate of approximately 2–3% [38, 65]. COVID-19 primarily transmits from person to person in a closed environment due to reduced air ventilation [57], the lack of ultraviolet light which can inactivate the virus and a reduced dilution indoors when compared to outdoor air [17]. Male sex, advanced age, underlying disease and comorbidities may be associated with severe illness and a higher rate of mortality [7]. In addition, COVID-19 appears to be correlated to the increasing rate of thromboembolic events in hospitalized patients [17, 37, 53].

Many factors contribute to disease emergence, including climate change, globalization and urbanization; most of these factors are caused by humans [23]. Anthropogenic activities are a major issue of air pollution due to the emission of harmful pollutants and sources of transmissible disease agents [5, 22–24, 43, 44, 73]. Fine particles, with an aerodynamic diameter lower than 2.5 µm (PM_2.5_) or 10 µm (PM_10_), are mainly emitted from sources such as vehicles, energy industries and dust [35, 43] and have potentially the most significant effects on human health when compared to other air pollutants [25]. Particles, especially PM_2.5_, are known to be responsible for different lung diseases and respiratory infections [45, 50, 56]. Previous studies showed that acute exposure to air pollutants increased the severity and the risk of hospital admissions for respiratory viral infections [28]. A good correlation was observed between air pollution and SARS-CoV-1 outbreak in China, furthermore, exposure to air pollution was shown to increase the transmission of viral infections [14, 18]. The transmission rate of SARS-CoV-2 could be affected by air pollution level, air temperature and relative humidity [27]. A study in Bangladesh indicated that high air temperature and relative humidity significantly reduced the transmission of COVID-19, while a peak of COVID-19 spread was observed at a mean temperature of 26 °C [36]. Some studies have reported a correlation between the spread of COVID-19, air pollution and some meteorological parameters [27, 36, 38, 54, 58, 66, 72, 79]. For the first time, this study investigated the relationships and time lag effects between PM_10_ and PM_2.5_ concentrations, daily hospital admissions for COVID-19, chest CT scans, air temperature and relative humidity in Ahvaz, Iran from March 2020 to March 2021.

## Materials and Methods

### The Study Area

**Fig. 1:**
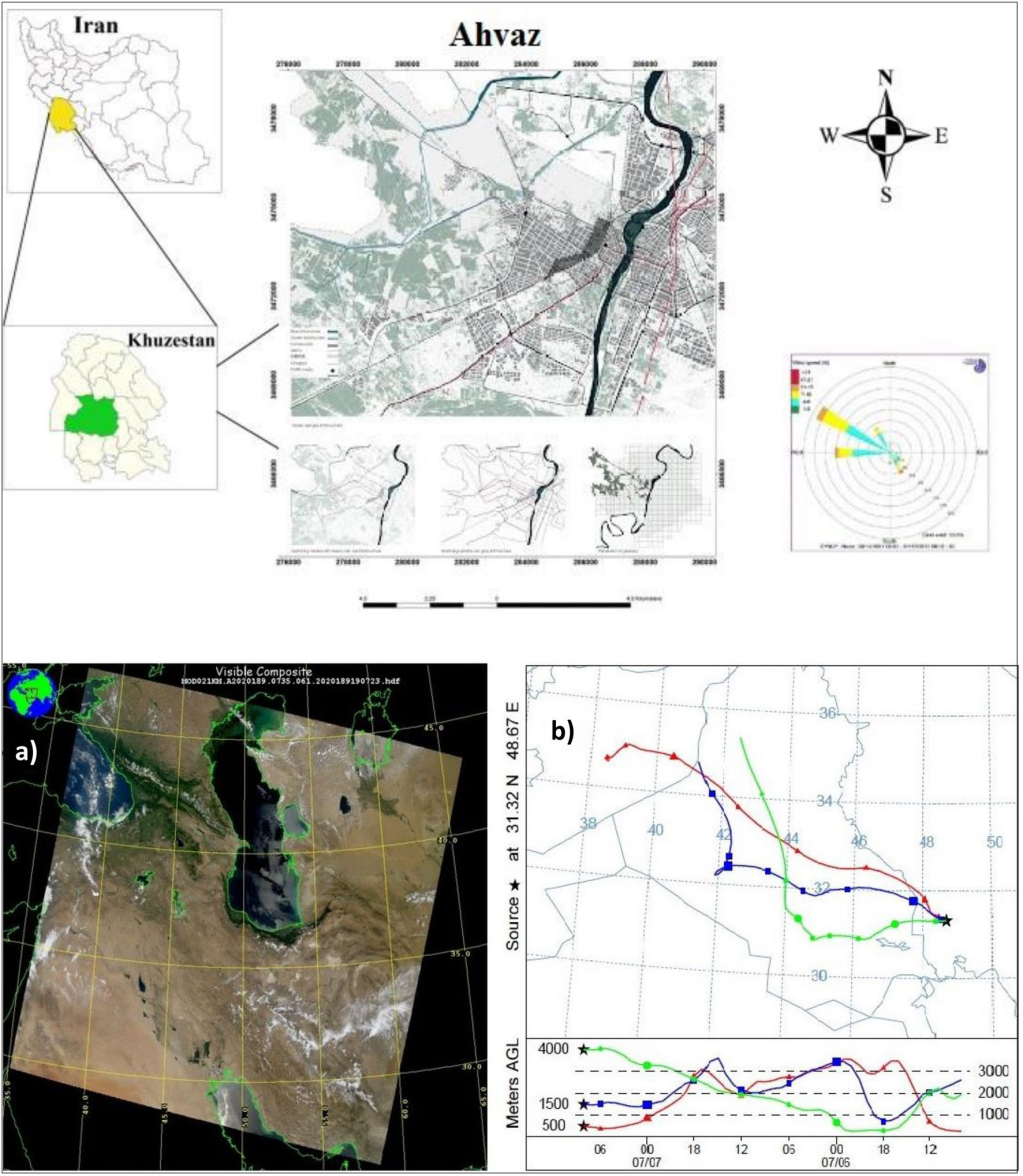
The location of study area, **a** MODIS of Ahvaz over a Middle Eastern dust storms on summer 2021 and **b** backward trajectory of particulate matters

Ahvaz (31° 19′ N 48° 40′ E), the capital city of Khuzestan province, is located in the southwest of Iran. This city covers an area of 185 km^2^ and has approximately 1.2 million inhabitants [6, 33, 40]. Ahvaz experiences a hot desert climate with a long summer and short winter. The annual mean temperature is approximately 24.9 °C and sand and dust storms are common [34]. Iranian cities are ranked as the most polluted by PM_10_ in the world [59, 72] and can be considered as a case study to investigate the effects of lockdown on PM levels. Figure 1 shows a map of Ahvaz and the Moderate Resolution Imaging Spectroradiometer (MODIS) of the city over Middle Eastern dust storms during summer 2021 with a backward trajectory of particulate matters (PM). Based on the annual PM_10_ mean concentration, Ahvaz (372 µg m^−3^ as the annual average in 2009) is the most polluted city in the world [55]. The high levels of PM in the air can be explained by industries located inside and around the city such as steel, gas and petroleum companies, oil refineries, and storms originating from the desert areas of Arabian countries [34].

### COVID-19 Data

An Acute Respiratory Section (ARS) or Respiratory Emergency (RE) unit was created in the Emergency Service at the Ahvaz Petroleum Hospital, located at the north of Ahvaz, for new daily cases with COVID-19 symptoms. Samples were taken by polymerase chain reaction (PCR); to evaluate chest symptoms, we used computed tomography scans (CT scan). Daily samples were recorded by the official group in the ARS and then the results were reported by the laboratory to the statistical team at the Emergency Operations Center (EOC). The results were recorded daily, including date, first name, surname, age, sex, PCR test result and chest CT scan, and if need be, fatality date. Patients who were referred for chest CT scans were screened for COVID-19 symptoms, and symptomatic patients were supported by a surgical mask and placed in an isolation room. All hospital referral data were taken from the Research and Education Center of Petroleum Hospital. More than 15,000 positive PCR and 10,400 positive chest CT scans were analyzed during the study period. We retrieved all data from 21 March 2020 until 20 March 2021.

### Environmental Data

Air quality monitoring data were provided by Ahvaz's Environmental Protection Agency (A-EPA). Due to obsolete or incomplete observations for several air pollutants, we restricted our regional analysis to two major air pollutants: hourly PM_10_ and PM_2.5_ concentrations (µg m^−3^) (http://khzdoe.ir/rha). Additional meteorological parameters, including hourly air temperature and relative humidity in Ahvaz from 21 March 2020 to 20 March 2021 were provided by the Meteorological Organization for four monitoring stations located in Ahvaz. Only background monitoring stations with at least 75% of validated hourly data in a year were included to calculate valid daily averages [42].

### Air Quality Related to PM_10_, PM_2.5_ and Lockdown

By using daily PM_10_ and PM_2.5_ concentrations, we investigated PM_10_ versus PM_2.5_ and the ratio PM_2.5_/PM_10_. To consider the short-term effects of meteorological parameters [72], the PM_10_ and PM_2.5_ mean concentrations during the COVID-19 pandemic (2020–2021) were compared to previous years (2015–2019). We defined three time periods: before (21 February to 21 March 2020), during (22 March to 21 April 2020) and after lockdown (22 April to 21 May 2020). In addition, the air quality index (AQI) was calculated.

$$\mathrm{AQI }= \frac{{\mathrm{IN}}_{\mathrm{HI}}-{\mathrm{IN}}_{\mathrm{LO}}}{{\mathrm{B}}_{\mathrm{HI}}-{\mathrm{B}}_{\mathrm{LO}}} \times \left(\mathrm{C}- {\mathrm{B}}_{\mathrm{LO}}\right)+ {\mathrm{IN}}_{\mathrm{LO}},$$

where, IN_HI_ and IN_LO_ represent the AQI value, B_HI_ and B_LO_ are breakpoint concentrations more than or less than C, and C is the concentration of each pollutant. This parameter can be divided into six categories including good, satisfactory, moderate, poor, very poor, and severe depending on whether the AQI respectively falls between 0–50, 51–100,101–200, 201–300, 301–400, and 401–500.

### Statistical Analyses

Data were tested for normality with the Kolmogorov–Smirnov one-sample *D* test. The non-parametric Spearman test was used to analyze correlations between variables and applied to daily data: PM_10_, PM_2.5_, temperature, relative humidity, COVID-19 cases, and positive chest CT scans. Statistical significance was set at *P* < 0.05 and statistical analyses were performed with Excel.

## Results

### Hospital Admissions Information

Of the 15,280 COVID-19 cases admitted to the Petroleum Hospital from 21 March 2020 to 20 March 2021 (Table 1), 57% of patients were male with an average age of 50.1 years; the average age of women was slightly higher (50.5 years). Among those with a positive PCR result, 40.0% of males and 46.8% of females experienced underlying diseases. With regards to the number of deaths, 56.6% were male; the average age of deaths for both male and female was similar (66.5 years).

**Table 1:** Referral information to the Petroleum Hospital between March 2020 and March 2021

	Male	Female
Percentage (%)	57.5	42.5
Average age (years)	50.1	50.5
People with underlying disease (%)	40.0	46.8
COVID-19 fatality rate (%)	56.6	43.4
Average age of deaths (years)	66.5	66.5

The highest number of patients were with the age ranges of 35–39 and 55–59 years, 12.3% and 11.3% of the total number of hospital admissions, respectively. Children (< 9 years) represented 0.1% of the total number of patients. Analysis showed that 61.7% of deaths by COVID-19 occurred in subjects over 65 years; 2.4% of people < 30 years of age died in Petroleum Hospital. The highest number of fatalities occurred in subjects who were 70–79 years of age. Furthermore, 35.9% of patients died in the 30–64 years age range (Table 2).

**Table 2:** Age groups of patients admitted to the Petroleum Hospital between March 2020 to March 2021

Age group	≤ 9	10–19	20–29	30–34	35–39	40–44	45–49	50–54	55–59	60–64	65–69	70–79	≥ 80
Referrals (%)	0.1	2.7	6.2	7.3	12.3	10.2	8.3	10.0	11.3	10.0	10.4	8.0	2.9
Mortality (%)	0	0	2.3	0.3	1.7	3.4	2.7	3.7	10.8	13.2	20.0	25.1	16.6

### Time-Series Variations of PM_10_and PM_2.5_

**Fig. 2:**
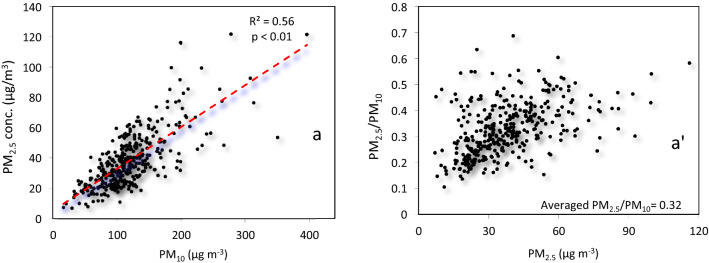
**a** Correlation between daily PM_10_ and PM_2.5_ (*P* < 0.01) concentrations and (**a**') PM_2.5_/PM_10_ ratio in Ahvaz (Iran) between March 2020 and March 2021

The highest daily PM_10_ and PM_2.5_ concentrations were observed on 18 March 2021 (396.4 µg m^−3^) and on 6 May 2020 (121.6 µg m^−3^), respectively (Fig. S1). In Ahavz, the highest (160.84 and 67.68 µg m^−3^) and the lowest (51.27 and 25.79 µg m^−3^) monthly PM_10_ and PM_2.5_ mean concentrations were also observed in October 2020 for PM_10_, and in January 2021 for PM_2.5_ (Fig. S2). The annual mean concentrations of PM_10_ and PM_2.5_ were 119.6 and 38.2 µg m^−3^, respectively, showing that PM_10_ and PM_2.5_ concentrations exceeded the annual limit value established by the WHO Air Quality Guideline for the protection of human health (50 and 25 µg m^−3^, respectively). A significant correlation between the daily mean concentrations of PM_10_ and PM_2.5_ was identified (*r* = 0.75, *P* < 0.01), with an average PM_2.5_/PM_10_ value of.0.32 (Fig. 2a, a' Table 3).

**Table 3:** Correlation coefficient (Spearman’s test) and significance level (*P*) between daily PM_10_ and PM_2.5_ mean concentrations, air temperature, relative humidity, the number of positive chest CT scan and COVID-19 cases at lag0

	PM_10_	PM_2.5_	T	RH	CT scan	COVID-19
PM_10_	1					
PM_2.5_	0.75, *P* = 0.004	1				
T	0.30, *P* = 0.304	− 0.20, *P* = 0.517	1			
RH	− 0.54, *P* = 0.05	0.09, *P* = 0.776	− 0.81, *P* = 0.018	1		
Positive CT scan	0.59, *P* = 0.031	0.56, *P* = 0.036	0.32, *P* = 0.160	− 0.82, *P* = 001	1	
COVID-19	0.47, *P* = 0.026	0.41, *P* = 0.044	− 0.33, *P* = 0.036	− 0.35, *P* = 0.045	0.71, *P* = 0.028	1

*T* temperature, *RH* relative humidity

### Correlation Analyses Between PM_10_, PM_2.5_, COVID-19 Cases and Positive Chest CT Scans

**Fig. 3:**
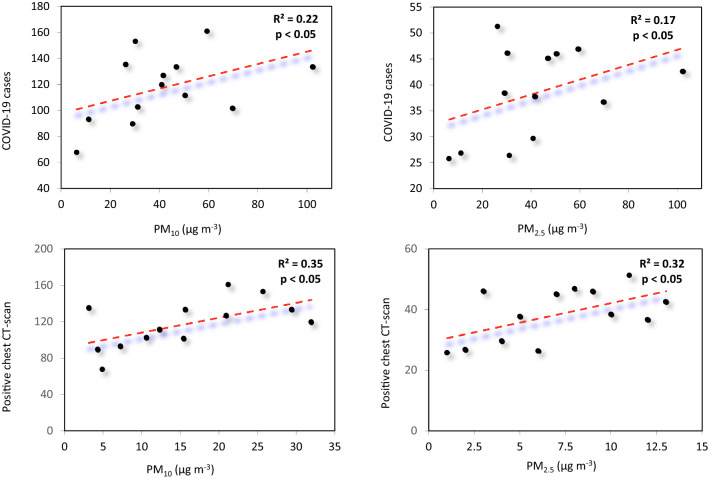
**a**, **a**' Correlation between the number of.COVID-19 cases admitted to the Petroleum Hospital with mean **a** PM_10_ and **a'** PM_2.5_, and **b**, **b'** correlation between the positive chest CT scan with mean **b** PM_10_ and **b'** PM_2.5_ during March 2020 to March 2021 in Ahvaz, Iran

The temporal pattern of monthly PM_10_ and PM_2.5_ *versus* COVID-19 cases and positive chest CT scans showed that with an increase of PM_10_ and PM_2.5_ concentrations, the rate of COVID-19 cases and positive chest CT scans also increased, especially from October 2020 to March 2021. The highest and lowest monthly average COVID-19 (79.36, 21.4 cases) and positive chest CT scans (31.93, 3.15 cases) were in March 2021 and April 2020 for COVID-19 with PM_10_ and PM_2.5_ respectively (124.45 and 93.15 µg m^−3^) and on June 2020 and January 2021 for positive chest CT scans with PM_10_ and PM_2.5_ concentrations of 31.93 and 3.15 µg m^−3^, respectively (Fig. S3). The PM_10_ and PM_2.5_ concentrations were correlated with the number of COVID-19 cases: *r* = 0.47 (*P* < 0.05), and *r* = 0.41 (*P* < 0.05), respectively (Fig. 3a–b', Table 3). Furthermore, the PM_10_ and PM_2.5_ concentrations were both correlated with positive chest CT scans (*r* = 0.59, *P* < 0.05, and *r* = 0.56, *P* < 0.05 respectively).

### Temporal Variations of Temperature and Relative Humidity

**Fig. 4:**
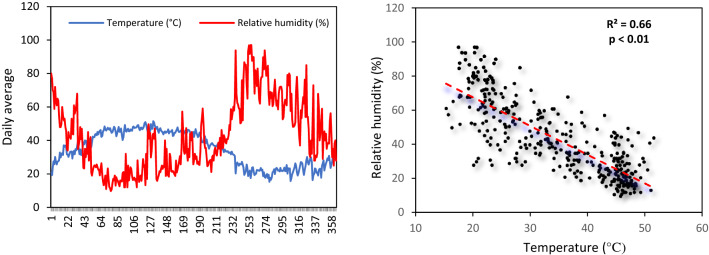
Trend daily variations of **a** air temperature and relative humidity and **a'** correlation between daily temperature and relative humidity during March 2020 to March 2021 in Ahvaz, Iran

The relationship between air temperature (°C) and relative humidity (%) are shown in Fig. 4. The highest daily temperature and relative humidity were 51.5 °C and 97%, respectively, on 30 July 2020 and 2 December 2020. The daily times series showed that an increasing temperature led to reduced relative humidity reaching a peak during summer for temperature and winter for relative humidity. Furthermore, the monthly variations showed that July 2020 with a mean air temperature of 47.8 °C and a relative humidity of 27% was the warmest during the time period of this study. The highest monthly average relative humidity 80% was identified in December 2020 with a mean temperature of 20.3 °C (Fig. S4). Multivariate analysis showed that the mean temperature was significantly associated with relative humidity (Table 3). The interaction between air temperature was negatively correlated (*r* =  − 0.81, *P* < 0.01) with relative humidity (Fig. 4a, a', Table 3).

### Meteorological Parameters, COVID-19 Incidence and Positive Chest CT Scans

The highest number of declared COVID-19 cases was observed when the daily air temperature and relative humidity ranged between 10.3 and 28.0 °C and between 19 and 40% respectively in February–March 2021. The lowest number of COVID-19 cases was registered when the relative humidity exceeded on average 60% with a mean air temperature of 20–30 °C in Ahvaz. The lowest average number of positive chest CT scans was observed for relative humidity ranged from 65 to 80% with 3.1–4.3 cases on average. The highest positive chest CT scan was obtained in June 2020 and March 2021 for daily air temperature ranging from 38 to 49 °C and 11 to 15 °C, respectively. In these two peaks, relative humidity ranged between 10–25% and 19–30%, respectively. Higher relative humidity (> 60%) strongly reduced the number of positive chest CT scan (see Fig. S5).

**Fig. 5:**
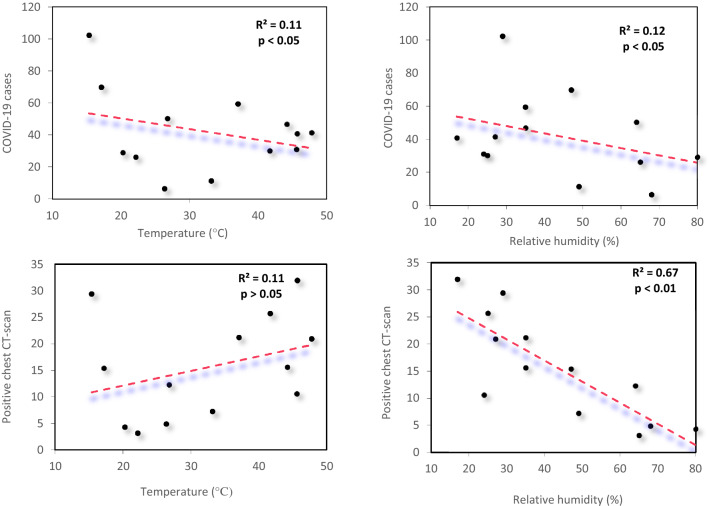
Correlation between average **a** air temperature and **a'** relative humidity with new cases of COVID-19 admitted to the Petroleum Hospital, and **b**, **b'** positive chest CT scan in Ahvaz between March 2020 and March 2021

The results of correlation between air temperature and relative humidity with COVID-19 cases and positive chest CT scan of patients admitted to the Petroleum Hospital during March 2020 to March 2021 are shown in Fig. 5a–b'. A negative correlation was found between COVID-19 incidence with air temperature (*r* =  − 0.33, *P* < 0.05) and relative humidity (*r* =  − 0.35, *P* < 0.05). There was no association between air temperature and positive chest CT scans (*r* = 0.32, *P* > 0.05) while the relative humidity and positive chest CT scans were negatively correlated (*r* =  − 0.82, *P* < 0.01).

### Time Lag Effect

A higher correlation (*r* = 0.60) was identified between daily PM_10_ concentrations at day + 10 and the number of COVID-19 cases at day 0 (Table 4), while the highest correlation with positive chest CT scans was observed on day 0 (*r* = 0.59). For daily PM_2.5_, the highest correlation with the number of COVID-19 cases was found between lag3 (*r* = 0.42) and lag0 with positive chest CT scans (*r* = 0.56). For air temperature and relative humidity, the highest correlations were found on day 0 (lag0).

**Table 4:** Correlation coefficient (Spearman’s test) between daily PM_10_ and PM_2.5_ mean concentrations, air temperature, and relative humidity with positive chest CT scan and COVID-19 cases at different time lags (0–13 days)

	PM_10_ vs COVID	PM_10_ vs CT scan	PM_2.5_ vs COVID	PM_2.5_ vs CT scan	T vs COVID	T vs CT scan	RH vs COVID	RH vs CT scan
lag0	0.47	**0.59**	0.41	**0.56**	** − 0.33**	**0.32**	** − 0.35**	** − 0.82**
lag1	0.35	0.55	0.30	0.52	− 0.24	0.30	− 0.23	− 0.63
lag2	0.50	ns	0.40	ns	− 0.26	0.28	− 0.22	− 0.63
lag3	0.55	ns	**0.42**	ns	− 0.24	0.28	− 0.20	− 0.62
lag4	0.43	ns	0.40	ns	− 0.22	0.29	− 0.22	− 0.63
lag5	0.47	ns	0.24	ns	− 0.22	0.28	− 0.21	− 0.61
lag6	ns	ns	ns	ns	− 0.21	0.27	− 0.23	− 0.61
lag7	ns	ns	ns	ns	− 0.23	0.27	− 0.27	− 0.60
lag8	ns	ns	ns	ns	− 0.22	0.28	− 0.21	− 0.58
lag9	ns	ns	ns	ns	− 0.23	0.27	− 0.21	− 0.60
lag10	**0.60**	ns	ns	ns	− 0.22	0.28	ns	− 0.60
lag11	0.47	ns	ns	ns	ns	0.28	ns	− 0.59
lag12	0.55	ns	ns	ns	ns	0.26	− 0.20	− 0.58
lag13	ns	ns	ns	ns	ns	0.26	− 0.22	− 0.55

The strongest correlations are shown in bold (*ns* not significant, *P* > 0.1)

### Air Quality and the Effect of Lockdown

**Fig. 6:**
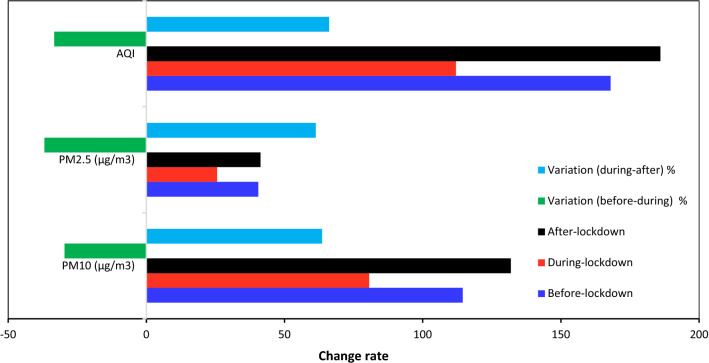
Mean PM_10_ and PM_2.5_ concentrations and air quality index (AQI) before (21 February to 21 March 2020), during (22 March to 21 April 2020) and after lockdown (22 April to 21 May 2020) in Ahvaz, Iran

The mean PM_10_ and PM_2.5_ concentrations and the AQI were calculated before, during and after lockdown from 21 February to 21 May 2020 (Fig. 6). Before the lockdown, the mean PM_10_ and PM_2.5_ concentrations and the AQI were 114.5 and 40.5 µg m^−3^ and 168, respectively. The reduction during the lockdown period (established from 22 March to 21 April 2020) was 29.6%, 36.9% and 33.3%, respectively. From 22 April 2020 to 21 May 2020, the AQI in Ahvaz increased by 66.1%; this was similar to the PM_10_ (+ 63.6%) and PM_2.5_ (+ 61.3%) concentrations.

A comparison of PM_10_ and PM_2.5_ mean concentrations between the pre-COVID-19 pandemic from 2015 to 2019 was carried out between 2020 and 2021 (Table 5). During the COVID-19 period, the annual PM_10_ and PM_2.5_ mean concentrations were reduced by 27.3% and 17.8%, respectively, when compared to the 5-years time period.

**Table 5:** Annual PM_10_ and PM_2.5_ mean concentrations during the pre-pandemic period (2015–2019) and during the COVID-19 period (2020–2021) in Ahvaz

Years	Average concentration	
	PM_10_ (µg m^−3^)	PM_2.5_ (µg m^−3^)
2015	167.2 ± 13.0	49.1 ± 48.5
2016	178.3 ± 21.2	50.7 ± 68.6
2017	159.2 ± 27.4	44.3 ± 33.2
2018	161.0 ± 96.1	44.8 ± 57.1
2019	155.8 ± 56.0	43.5 ± 12.8
**2015–2019**	**164.5 ± 80.9**	**46.5 ± 44.0**
**2020–2021**	**119.6 ± 24.0**	**38.2 ± 37.1**

## Discussion

In the Middle Eastern regions, the annual PM_10_ and PM_2.5_ mean concentrations typically ranged from 72 to 303 and 11 to 35 µg m^−3^, respectively [34]. Over the 2015–2019 period, the annual PM_10_ mean concentrations ranged from 155.8 to 178.3 µg m^−3^ while the PM_2.5_ mean concentrations ranged from 43.5 to 50.7 µg m^−3^. In 2020–2021, during the COVID-19 pandemic, the annual PM_10_ and PM_2.5_ mean concentrations were 119.6 and 38.2 µg m^−3^, respectively. The poor and continuous deterioration in air quality in Ahvaz is due to the presence of several industries inside and around the city and dust storms from Arabian countries [4, 20, 41, 73]. The PM_2.5_/PM_10_ ratio in the Middle East is due to the high levels of PM during dust storms and ranges from 0.21 to 0.59 [42]. Our analysis revealed a PM_2.5_/PM_10_ of 0.32 in Ahvaz from March 2020 to March 2021. Depending on the fraction of coarse dust in the air, this ratio may undergo change [29, 42]. This low value is typical of regions dominated by geological dust in contrast to rural areas where combustion processes involving coal or wood burning show predominance [30].

Approximately 61.7% of deaths by COVID-19 occurred in subjects over 65 years of age in Petroleum Hospital and only 2.4% of deaths occurred in younger subjects (those < 30 years of age). Reference [61] showed that 80% of deaths by COVID-19 occurred in adults over 65 years of age in the United States and concluded that most deaths occurred in the 60–69-year age group in five European countries (Italy, Spain, France, Germany and the Netherlands). In Italy, the fatality rates ranged from less than 3% in subjects < 60 years of age to more than 30% in people > 80 years of age [74]. The large differences in fatality rates by age are associated to higher rates of chronic comorbidities in elder populations [74, 75]. Our study confirms previous results [17] which reported that males were more at risk of COVID-19. Preliminary studies were performed to assess the effects of air pollution (e.g., PM_10_, PM_2.5_) and meteorological parameters (e.g., air temperature, relative humidity) on the spread of COVID-19 [48, 82]. We are still far from fully understanding COVID-19 epidemiology, so conducting different studies could help enhance our understanding of this disease.

Epidemiological studies have confirmed the association of PM with respiratory diseases and found that fine PM could potentiate viral transmissions [48, 73, 78]. Furthermore, viral replication in the respiratory system is enhanced by the negative effects of PM on the integrity of the human respiratory barrier [48]. The influenza and respiratory syncytial viruses remain in the air for a long period after becoming attached to PM,this allows viruses to be transmitted by the airborne route [51]. In this study, both the PM_10_ and PM_2.5_ concentrations were positively correlated with the incidence of COVID-19 (chest CT scans and PCR tests). The incidence of COVID-19 increased with rising PM_10_ and PM_2.5_ concentrations [82] and a high frequency of PM_10_ concentration peaks (exceeding 50 μg m^−3^) resulted in an accelerated spread of COVID-19, thus suggesting a “boost effect” with regards to viral infectivity [71]. The increase in outdoor air PM_10_ and PM_2.5_ concentrations was positively associated with an increased risk of COVID-19 transmission [9]. Reference [48] reported a significant correlation between PM_10_ (*r*^2^ = 0.15), PM_2.5_ (*r*^2^ = 0.23) and the incidence of COVID-19 between the 26th of January and the 29th of February 2020 in Wuhan and XiaoGan (China).

A 1 µg m^−3^ increase in long-term PM_2.5_ exposure is related to a 15% increase in the risk of death by COVID-19 [79]. Reference [81] reported a positive association between the incidence of COVID-19 and both PM_2.5_ (*r* = 0.48) and PM_10_ (*r* = 0.49) concentrations in Wuhan (China). Following an increase of 10 µg m^−3^ in PM_10_ and PM_2.5_, the mortality rate of COVID-19 increased by 0.83% and 0.86%, respectively [81]. Similarly, in Europe, positive relationships were found between PM_10_, PM_2.5_, and the fatality rate of COVID-19 [26]. Other studies have reported that short-term PM_2.5_ exposure is positively correlated to the risk of respiratory infection by COVID-19 among people in Canada and New York [3, 76]. In the present study, higher correlations were found between PM_10_, PM_2.5_ and chest CT scans compared to PCR tests. In China, only 3% of admitted patients had negative PCR tests while having positive chest CT scans [80]. However, PCR tests are easy to perform and provide fast results, thus enabling the rapid diagnosis of COVID-19 [1].

The mean temperature and air quality were previously shown to be significantly associated with the COVID-19 pandemic in New-York [10]. Air temperature and relative humidity are the most important meteorological parameters affecting COVID-19 mortality and the air temperature is known to be correlated to the spread of COVID-19 [16, 54, 63]. In this study, we also found that high daily air temperature (30–51 °C) and high relative humidity (60–97%) led to a significant reduction in the daily incidence of COVID-19. High air temperature and relative humidity appear to have an inhibitory effect on COVID-19 transmission. The virus was transmitted more efficiently in Ahvaz at a daily air temperature of 10–28 °C with a relative humidity of 19–40%. In Milan (Italy), [82] showed there was a negative association between daily air temperature and relative humidity with the number of new cases of COVID-19. In Wuhan, the daily temperature (*r*^2^ = 0.14) was inversely correlated to the incidence of COVID-19 [48]. Reference [8] reported that cities with a mean air temperature lower than 24 °C were all at high-risk of COVID-19 transmission while [13] showed that the most suitable air temperature for the spread of coronavirus was 13–24 °C with a relative humidity of 50%. Our findings are in line with this previous research.

Lockdown due to the COVID-19 pandemic reduced industrial activities, transport and energy consumption [38]. In Iran, 21 March to 21 April 2020 was selected as a period of lockdown by the government to reduce the incidence of COVID-19. During the lockdown, there was a significant reduction in PM_10_ (29.6%), PM_2.5_ (36.9%), and AQI (33.3%) in Ahvaz when compared to the month before (21 February to 21 March 2020). During the COVID-19 pandemic, the annual mean concentrations of PM_10_ (27.3%) and PM_2.5_ (17.8%) were reduced when compared to the previous 5-year period (2015–2019) [38] also showed that PM_10_ and PM_2.5_ concentrations and AQI decreased by 15%, 8% and 13%, respectively, during the lockdown in Baghdad. Reference [11] reported that in Malaysia, PM_10_ and PM_2.5_ concentrations decreased during lockdown by 26–31% and 23–32%, respectively. This significant reduction of PM levels was widely observed in Europe, the United States and in Asia before and during the lockdown (e.g., Andrée, 2020; Masum and Pal, 2020) [72]. By considering temporal data, trends analysis in Marseille (southern France) showed that the restrictive measures due to COVID-19 led to 11% reduction in PM_2.5_ concentrations when compared to data for 2010 to 2019 [42]. A comparison between different studies and the variations in PM_10_ and PM_2.5_ concentrations during COVID-19 lockdown is presented in Table 6.

**Table 6:** The results from studies describing the variations of PM_10_ and PM_2.5_ during the COVID-19 lockdown

Study location	Methodology	Variation %		Main point	References
		PM_10_	PM_2.5_		
Delhi	Three stations in Delhi were selected at three periods from 1 January to 24 March	− 63.5	− 69.4	Due to decrease in human activities and social distances, the PM_10_ and PM_2.5_ concentrations were decreased	[31]
Salé city	PM_10_ before (11–20 March) and during (21 March 21 to 2 April) lockdown was measured	− 75	−	The emissions rate from vehicle exhaust and industrial activities were significantly reduced in this time, which contribute to the decrease in the concentrations of particles	[62]
Valencia	The percentage change at lockdown and same time over 2017–2019 was assessed	− 13	− 32	PM_10_ and PM_2.5_ were decreased at Valencia during COVID-19 lockdown due to reduction in traffic and fuel combustion	[72]
Guangdong	Data from 101 air quality monitoring sites were collected for comparison among before and during lockdown	− 51.5	− 37.8	All considerable data PM_10_, PM_2.5_, O_3_, and NO_2_ were reduced by change in human-made impacts. After lockdown the NO_2_ concentration was remarkably increased	[49]
Kolkata	Two years data 2019 and 2020 were obtained from State Pollution Control Board, as well as trajectory analysis was done by HYSPLIT	− 36.7	− 43.7	In comparison with 2019, the PM_10_ and PM_2.5_ were declined It is clear lockdown plays a significant role to reduce particles concentration	[12]
Bengaluru	Monthly air pollution concentration in March, April, and May 2020 were compared with that of 2019 to clarify the effect of restricted emissions	− 40.2	− 33.9	Due to restriction on anthropogenic activity, emission sources were declined, thus the PM concentration was reduced at Bengaluru	[46]
Hanoi	The data of pre-lockdown (10–31 March) and lockdown (1–22 April) were analyzed to the assess of variation in pollutants concentrations	–	− 55	Results showed that a drastic negative relationship was observable between the boundary layer height and the daily mean PM_2.5_ from industrial activities	[60]
Seoul	A comparison between data 2020 with three years ago	− 25.4	− 36	The comparison with the previous three years showed a drop in PM. The rapid decline in the traffic (30% to 70%) led to an improvement in air quality	[70]

In this study, PM_2.5_ and PM_10_ concentrations were averaged from air quality monitoring stations. We also assumed that all individuals shared the same particle levels in and around Ahvaz; therefore the individual exposure estimate was erroneous [72]. In addition, only the outdoor exposure to PM was considered while people spent almost 100% of their time in indoor environments during lockdown; PM concentrations are generally lower indoors than outdoors [47]. A high concentration on a given day can produce effects over the following days, i.e., a lag effect, and accumulative exposure can produce larger delayed effects [67]. The estimated effects are erroneous by omitting the lag effect of PM exposure, i.e., by assuming that all effects occur on one day [69]. Lag models with la ag of several days/weeks have offered a better measure of the effects of air pollution on human health when compared to the single day model [2, 52]. Higher correlations were found between daily PM_2.5_ and PM_10_ concentrations and the number of COVID-19 cases at 3- and 10-days lag, while positive chest CT scans were better correlated to PM_2.5_ and PM_10_ with no lag. In Ahvaz, significant relationships were reported between an increase in PM_10_ levels and cardiovascular deaths between 3- and 13-days lag [21].

## Conclusion

In Ahvaz, males over 65 years of age were at high risk of COVID-19. In this study, several parameters were analyzed to provide additional evidence relating to the relationship between particle pollution (PM_10_ and PM_2.5_), meteorological parameters (air temperature and relative humidity) and COVID-19 incidence between May 2020 and May 2021 in Ahvaz (Iran). Significant correlations between daily PM_10_ and PM_2.5_ concentrations were identified. We found a positive relationship between PM_10_ and PM_2.5_ with COVID-19 incidence and positive chest CT scans, thus yielding further evidence that air pollution provides a favorable context for the spread of the SARS-CoV-2 virus [19, 82]. The interaction between daily air temperature was negatively correlated with relative humidity. In Ahvaz, an air temperature between 10 and 28 °C and a relative humidity between 19 and 40% were found to be most suitable for the spread of coronavirus. However, a high daily air temperature (> 30 °C) and relative humidity (> 60%) significantly reduced the incidence of COVID-19. Air quality was notably improved during the COVID-19 lockdown; this led to a large reduction in PM_10_, PM_2.5_ and AQI when compared to mean concentrations over the previous 5 years (2015–2019).

## Supplementary Information

Below is the link to the electronic supplementary material.

Supplementary file1 (DOCX 91 KB)

## Data Availability

Not applicable.

## Abbreviations

PM: Particulate matters
MODIS: Moderate Resolution Imaging Spectroradiometer
ARS: Acute Respiratory Section
RE: Respiratory emergency
PCR: Polymerase chain reaction
CT-scan: Computed tomography scan
EOC: Emergency Operations Center
AQI: Air quality index
