# Supplementary material for: COVID-19 Outbreak Related to PM10, PM2.5, Air Temperature and Relative Humidity in Ahvaz, Iran
Source: Dr. Sulaiman Al Habib Med J. 2022 Nov 30;4(4):182–95. doi: 10.1007/s44229-022-00020-z (PMC9713103; doi:10.1007/s44229-022-00020-z)
Supplement: Supplementary file 1 — Supplementary file1 (DOCX 91 KB) [file 44229_2022_20_MOESM1_ESM.docx]

**COVID-19 outbreak related to PM_10_, PM_2.5_, air temperature, and relative humidity in Ahvaz, Iran**

**Yusef Omidi Khaniabadi ^1^, Pierre Sicard ^2^, Bahram Dehghan ^3^, Hassan Mousavi ^3,4^, Saeid Saeidimehr ^3^, Mohammad Heidari Farsani ^1,*^, Sadegh Moghimi Monfared ^5^, Heydar Maleki ^6^, Hojat Moghadam ^1^, Pooran Molaei Birgani ^3^**

1. Occupational and Environmental Health Research Center, Petroleum Industry Health Organization (PIHO), Ahvaz, Iran
2. ARGANS, Sophia-Antipolis, France.
3. Family Health Research Center, Petroleum Industry Health Organization (PIHO), Ahvaz, Iran
4. School of Medicine, Ahvaz Jundishapur University of Medical Sciences, Ahvaz, Iran
5. Gachsaran Oil and Gas Production Company, National Iranian Oil Company, Gachsaran, Iran
6. Department of Environmental Health Engineering, School of Public Health, Student Research Committee, Ahvaz Jundishapur University of Medical Sciences, Ahvaz, Iran

* **Corresponding authors:** [hmohammad365@yahoo.com](mailto:hmohammad365@yahoo.com), heidarimfar@gmail.com

Fig. S1. Daily-averaged time series plot of PM_10_ and PM_2.5_ in Ahvaz (Iran) during the study period March 2020 to March 2021.


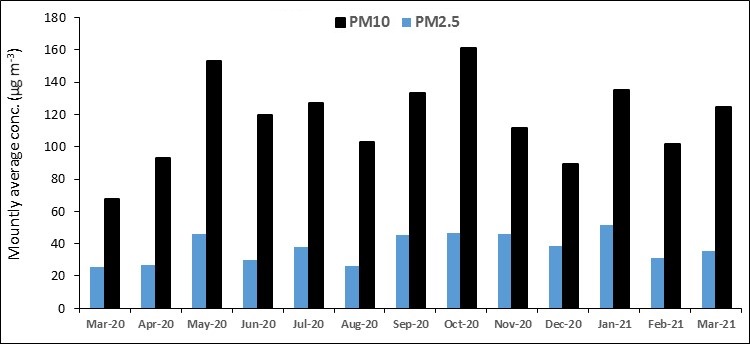


Fig.S2. Monthly-averaged mean concentration of PM_2.5_ and PM_10_ in Ahvaz (Iran) between March 2020 to March 2021.

Fig. S3. Association between PM_10_, PM_2.5_ with COVID-19 cases and positive chest CT-scan among people with symptoms in Ahvaz (Iran) between March 2020 to March 2021.

Fig. S4. Monthly-averaged temperature (°C) and relative humidity (%) in Ahvaz (Iran) between March 2020 to March 2021.

Fig.S5. Association between temperature (°C) and relative humidity (%) with COVID-19 cases and positive chest CT-scan among people with symptoms in Ahvaz (Iran) between March 2020 to March 2021.
